# Induction of Oxidative Stress and Mitochondrial Dysfunction by Juglone Affects the Development of Bovine Oocytes

**DOI:** 10.3390/ijms22010168

**Published:** 2020-12-26

**Authors:** Ahmed Atef Mesalam, Marwa El-Sheikh, Myeong-Don Joo, Atif Ali Khan Khalil, Ayman Mesalam, Mi-Jeong Ahn, Il-Keun Kong

**Affiliations:** 1 Division of Applied Life Science (BK21 Four), Gyeongsang National University, Jinju 52828, Korea; Am.mesalam@nrc.sci.eg (A.A.M.); Marwa.el-sheikh@hotmail.com (M.E.-S.); jmd1441@gmail.com (M.-D.J.); 2 Department of Therapeutic Chemistry, Division of Pharmaceutical and Drug Industries Research, National Research Centre (NRC), Dokki, Cairo 12622, Egypt; 3 Department of Microbial Biotechnology, Genetic Engineering and Biotechnology Division, National Research Centre (NRC), Dokki, Cairo 12622, Egypt; 4 Department of Biological Sciences, National University of Medical Sciences, Rawalpindi 46000, Pakistan; Atif.khalil7799@gmail.com; 5 Department of Theriogenology, Faculty of Veterinary Medicine, Zagazig University, Zagazig 44519, Egypt; Aymanmesalam@gmail.com; 6 College of Pharmacy and Research Institute of Pharmaceutical Sciences, Gyeongsang National University, Jinju 52828, Korea; Mjahn07@gmail.com; 7 Institute of Agriculture and Life Science, Gyeongsang National University, Jinju 52828, Korea; 8 Division of Applied Life Science (BK21 Plus), Gyeongsang National University, Jinju 52828, Korea

**Keywords:** juglone, ROS, apoptosis, cleavage, bovine, oocyte, blastocyst, IVM

## Abstract

Juglone, a major naphthalenedione component of walnut trees, has long been used in traditional medicine as an antimicrobial and antitumor agent. Nonetheless, its impact on oocyte and preimplantation embryo development has not been entirely clarified. Using the bovine model, we sought to elucidate the impact of juglone treatment during the in vitro maturation (IVM) of oocytes on their maturation and development of embryos. Results showed a severe reduction in oocyte nuclear maturation and cumulus expansion and a significant increase in mitochondrial dysfunction and reactive oxygen species (ROS) levels in cumulus–oocyte complexes (COCs) treated with juglone (12.5, 25.0, and 50.0 µM). In addition, RT–qPCR showed downregulation of the expansion-related (HAS2, TNFAIP6, PTX3, and PTGS2) and mitochondrial (ATPase6 and ATP5F1E) genes in juglone-treated COCs. Moreover, the development rates of day 4 total cleavage and 8–16 cell stage embryos, as well as day 8 blastocysts, were significantly reduced following exposure to juglone. Using immunofluorescence, the apoptotic marker caspase-9 was overexpressed in oocytes exposed to juglone (25.0 µM) compared to the untreated control. In conclusion, our study reports that exposing bovine oocytes to 12.5–50.0 µM of juglone can reduce their development through the direct induction of ROS accumulation, apoptosis, and mitochondrial dysfunction.

## 1. Introduction

Juglone is a phenolic compound identified in the roots, barks, and leaves of many Juglans (walnut trees) species comprising *J. nigra*, *J. regia*, and *J. cinerea,* as well as Proteaceae, Caesalpiniaceae, Fabaceae, and the hickory tree [1]. The cytotoxic properties of juglone (1.0–100.0 µM) have been reported in vitro against different cancer cells such as cervical, gastric, pancreatic, breast, prostate, lung, and ovarian cancer cells [1,2,3,4,5,6]. In addition, juglone exhibits antifungal (e.g., *Aspergillus* and *Penicillium*), antibacterial (e.g., *Escherichia coli*, *Staphylococcus aureus*, *Bacillus subtilis*, and *Helicobacter pylori*), antiviral, and antiparasitic activities [4,7,8]. However, juglone also suppressed lipopolysaccharides-induced inflammatory responses and NLRP3 inflammasome activation in tumor-derived J774.1 cells [9]. For these reasons, it has long been used in traditional medicine against various health conditions, including gastrointestinal disorders, cancers, allergies, and different microbial infections [6].

Oocyte maturation comprises the transfer of naturally arrested oocytes from the germinal vesicle (GV) stage to the metaphase II (MII) stage [10]. During this process, the oocyte acquires the ability to generate an activation response, triggered by sperm during fertilization, and initiate subsequent embryonic development [11,12]. The environmental conditions surrounding the oocyte play an essential role during the multiple developmental processes, including cumulus expansion, chromosomal condensation, and progression to MII-stage [13,14]. Additionally, mitochondria play an essential role in maintaining the functionality of oocytes by providing the main supply of ATP essential for maturation, fertilization, and embryo development [15,16].

The successive release of reactive oxygen species (ROS) is one of the main hurdles for the production of high-quality embryos. Under physiologic conditions, normal mitochondrial metabolism results in the generation of potentially damaging levels of ROS [17], which are mostly eliminated by the defense mechanisms comprising enzymes and free radical scavengers [18]. However, successive leakage of ROS from mitochondria into the cytoplasm, in response to adverse conditions such as illness, inflammation, and cytotoxicity, can induce damage to several cell components including DNA, proteins, and lipids, which can negatively affect the development of embryos and the pregnancy rate [19,20].

Over the past two decades, several biomolecules and antioxidants have been identified as enhancers of embryonic development via reducing ROS levels, whereas other molecules have proved to stimulate oxidative stress and hence impair embryonic development [12,21,22,23,24]. Moreover, the toxicity of juglone, reported in multiple studies, has been mainly attributed to its ability to induce endogenous ROS accumulation, DNA damage, autophagy, apoptosis, and inhibition of protein expression [2,25,26,27,28]. In addition, juglone is also able to inhibit the proliferation of lymphocytes through blockage of potassium channels and induction of plasma membrane polarization [29]. Despite the multiple pharmacological actions of juglone, only two recent studies have used juglone-vitamin C cotreatment during in vitro maturation (IVM) and post-fertilization of porcine and mouse oocytes, respectively, to show that vitamin C can protect cumulus–oocyte complexes (COCs) and presumptive zygotes from the detrimental effect of juglone [25,30].

Since the interplay between juglone and the different cellular processes regulating the oxidative stress and the developmental competence of bovine oocytes is not completely clarified, we sought to elucidate the safety of the use of therapeutical juglone on reproduction through investigating its impact on the IVM of oocytes, mitochondrial dysfunction, cleavage and blastocyst development rates, as well as the levels of ROS and apoptosis.

## 2. Results

### 2.1. High Juglone Concentrations Negatively Affect Cumulus Expansion

**Figure 1:**
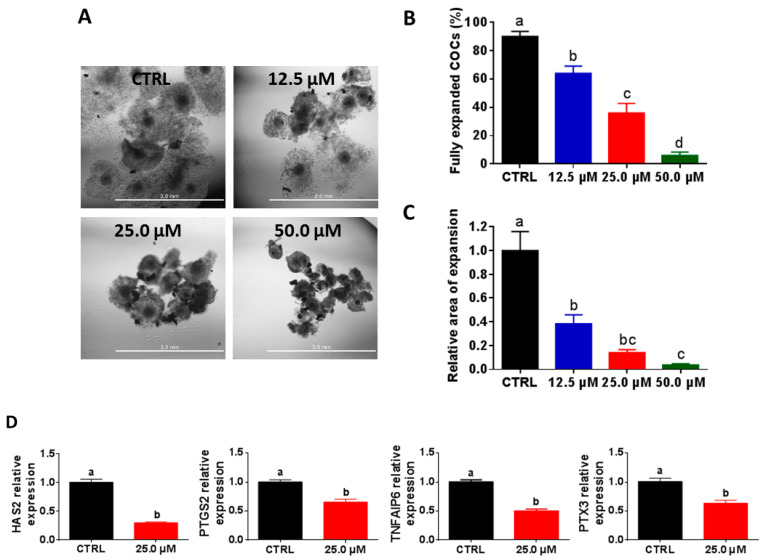
Cumulus expansion patterns following juglone treatment. (**A**) Morphological appearance of cumulus–oocyte complexes (COCs) after maturation. (**B**) The percentage of COCs with fully expanded cumulus cells. (**C**) The relative change in COCs expansion area. (**D**) Investigation of the relative expression of expansion-specific genes using RT–qPCR. Scale bar = 2.0 mm. Data are presented as mean ± SEM. The different letters on the columns reflect statistical significance (*p* < 0.05).

To clarify the impact of juglone administration on embryo development, we initially inspected the competence of cumulus cells expansion post-juglone treatment. In a dose–response experiment, cumulus–oocyte complexes (COCs) were co-incubated with three juglone concentrations (12.5, 25.0, and 50.0 µM) for 22 h. Checking COCs under the microscope at days 0 and 1 from the onset of culturing revealed that cumulus cells surrounding the majority of oocytes of the untreated group were fully expanded, whereas a significant reduction in the properly expanded cumulus cells was observed in the three juglone-treated groups in a dose-dependent manner (64.0 ± 5.09%, 36.0 ± 6.78%, and 6.0 ± 2.45% for 12.5, 25.0, and 50.0 µM juglone vs. 90.0 ± 3.54% for control; Figure 1A,B; *p* < 0.05). In addition, the relative area of expansion was significantly lower under juglone treatment compared to the untreated control (0.38 ± 0.07, 0.14 ± 0.03, and 0.04 ± 0.01 for 12.5, 25.0, and 50.0 µM vs. 1.0 ± 0.16 for control; Figure 1C; *p* < 0.05). For confirmation, the relative transcription levels of the cumulus expansion-specific genes TNF alpha-induced protein 6 (TNFAIP6), hyaluronan synthase 2 (HAS2), prostaglandin-endoperoxide synthase 2 (PTGS2), and pentraxin 3 (PTX3) were investigated using RT–qPCR. As seen in Figure 1D, the four tested genes were significantly downregulated in COCs exposed to 25.0 µM juglone compared to the control group (*p* < 0.05).

### 2.2. Reduction of Oocyte Maturation following Exposure to Juglone

**Figure 2:**
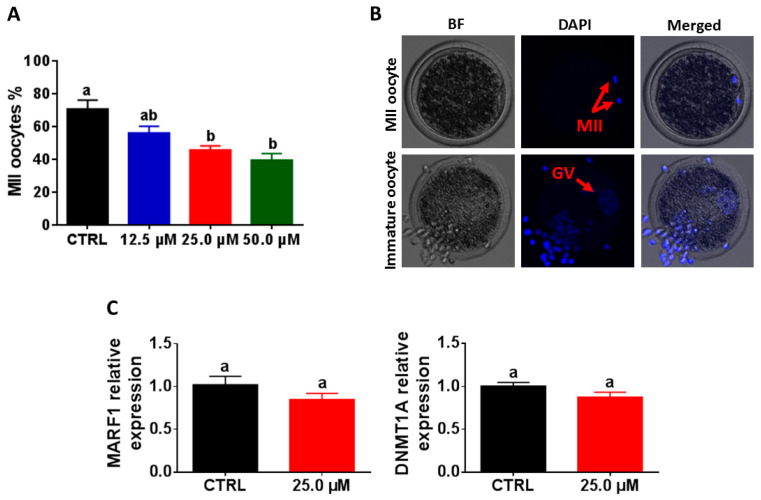
The effect of juglone on oocyte maturation. (**A**) The percentage of mature oocytes. (**B**) DAPI staining of nuclear material of MII and immature oocytes. (**C**) The expression pattern of maturation genes inspected by RT–qPCR. BF: bright field. Original magnification 100×. The different letters on the columns reflect statistical significance (*p* < 0.05).

For evaluating the maturation of oocytes following juglone treatment, the nuclear material was stained with DAPI and visualized under microscope 22 h from the onset of IVM. Similar to the expansion results, juglone treatment was associated with a significant decline in the percentage of oocytes that reached MII stage (56.3 ± 3.99%, 45.8 ± 2.43%, and 39.6 ± 3.98% for 12.5, 25.0, and 50.0 µM vs. 70.8 ± 5.38% for control; Figure 2A,B; *p* < 0.05). However, the use of RT–qPCR could not show significant differences in the expression pattern of the two maturation genes DNA methyltransferase 1 (DNMT1A) and the meiosis regulator and mRNA stability factor 1 (MARF1) between the treated and the control oocytes (Figure 2C; *p* > 0.05).

### 2.3. Juglone Administration during the IVM Reduces Embryonic Development

**Figure 3:**
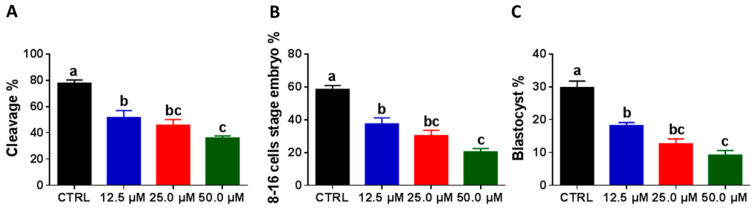
The developmental competence of embryos after juglone treatment. (**A**) Day 4 total cleavage. The development rates of 8–16 cell-stage (**B**) and day 8 embryos (**C**). The different letters on the columns reflect statistical significance (*p* < 0.05).

To scrutinize the effect of juglone on embryonic development, cleavage and blastocyst development rates were verified at days 4 and 8 post-fertilization, respectively. Interestingly, juglone supplementation reduced the day 4 total cleavage (51.6 ± 5.39%, 45.8 ± 4.28%, and 36.0 ± 1.52% for 12.5, 25.0, and 50.0 µM vs. 77.8 ± 2.38% for control; Figure 3A; *p* < 0.05) and 8–16 cell-stage rates (37.4 ± 3.68%, 30.4 ± 3.19%, and 20.4 ± 2.02% for 12.5, 25.0, and 50.0 µM vs. 58.4 ± 2.42% for control; Figure 3B; *p* < 0.05). Similarly, day 8 blastocyst development rates were also reduced in the groups developed from juglone-treated oocytes (18.2 ± 0.92%, 12.6 ± 1.50%, and 9.2 ± 1.39% for 12.5, 25.0, and 50.0 µM vs. 29.8 ± 1.93% for control; Figure 3C; *p* < 0.05).

### 2.4. High Juglone Concentrations Induce ROS Accumulation in Oocytes

**Figure 4:**
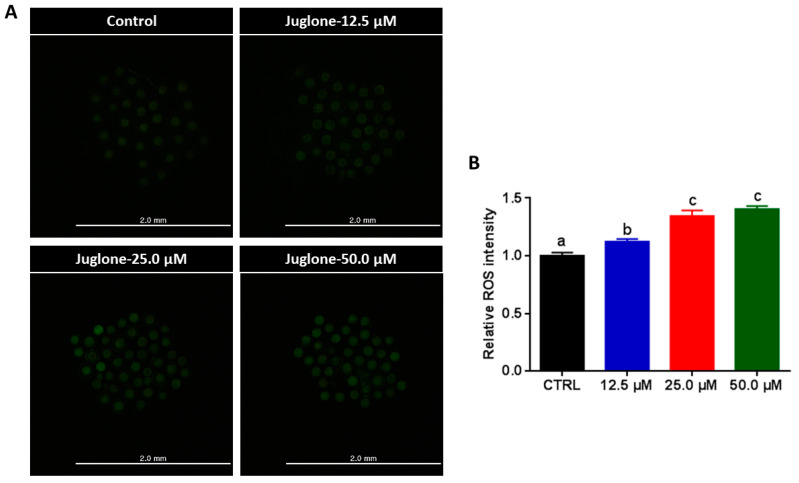
Intracellular reactive oxygen species (ROS) levels following juglone treatment. (**A**) 2,7-Dichlorodihydrofluorescein diacetate (H_2_DCFDA) staining of oocytes. (**B**) The relative ROS fluorescence intensity in the in vitro matured oocytes analyzed by ImageJ. Scale bar = 2.0 mm. The different letters on the columns reflect statistical significance (*p* < 0.05).

Next, the impact of juglone on oxidative stress was assessed after IVM via estimating the intracellular ROS levels using H_2_DCFDA-based labeling. As seen in Figure 4A,B, juglone treatment was associated with a significant increase in ROS levels in the entire cytoplasm of all juglone-treated oocytes in a dose-dependently manner (*p* < 0.05).

### 2.5. Juglone Affects the Distribution Pattern of Oocyte’s Mitochondria

**Figure 5:**
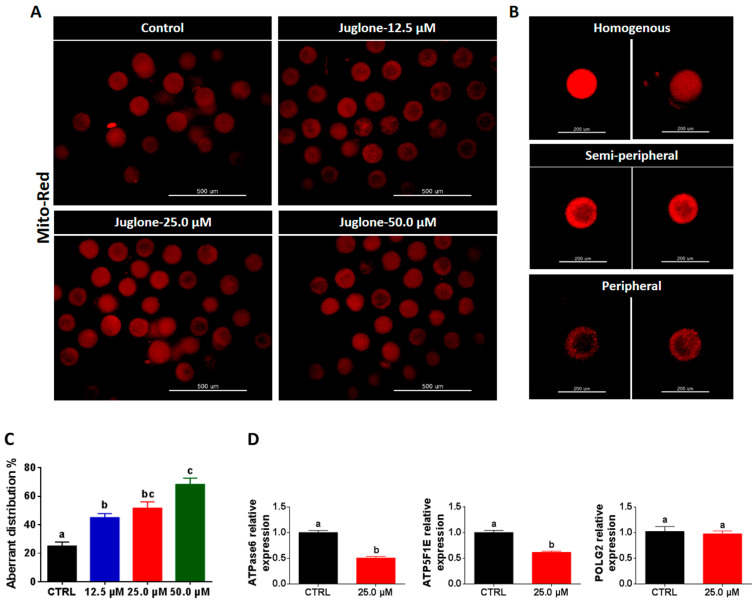
Mitochondrial distribution pattern after juglone administration. (**A**) Mito-Tracker Red labeling of mitochondria in juglone-treated and the control oocytes. Scale bar = 500 µm. (**B**) Representative images for the homogeneous and aberrant (semi-peripheral and peripheral) distribution patterns of mitochondria. Scale bar = 200 µm. (**C**) Percentage of oocytes with an aberrant pattern. (**D**) The relative expression of the mitochondrial genes ATPase6, ATP5F1E, and POLG2 tested using RT–qPCR. Mito-Red: Mito-Tracker Red. The different letters on the columns reflect statistical significance (*p* < 0.05).

In a similar experimental setting, the three different juglone concentrations were applied during IVM, whereas the mitochondrial distribution pattern was inspected after 22 h of treatment. Using Mito Tracker-Red staining, the majority of oocytes matured under juglone pressure exhibited aberrant (semi-peripheral and peripheral) distribution patterns (45.0 ± 2.89%, 51.7 ± 4.41%, and 68.3 ± 4.41% corresponding to 12.5, 25.0, and 50.0 µM juglone, respectively compared to 25.0 ± 2.88% of control; Figure 5A–C; *p* < 0.05). In line with these observations, the use of RT–qPCR showed significant downregulation of the mitochondrial genes ATP synthase F1 subunit epsilon (ATP5F1E) and ATP synthase membrane subunit 6 (ATPase6) in juglone-treated oocytes, while the expression pattern of DNA polymerase gamma 2 (POLG2) was statistically non-significant when compared to control (Figure 5D).

### 2.6. Juglone Treatment Induces Apoptosis in Oocytes

**Figure 6:**
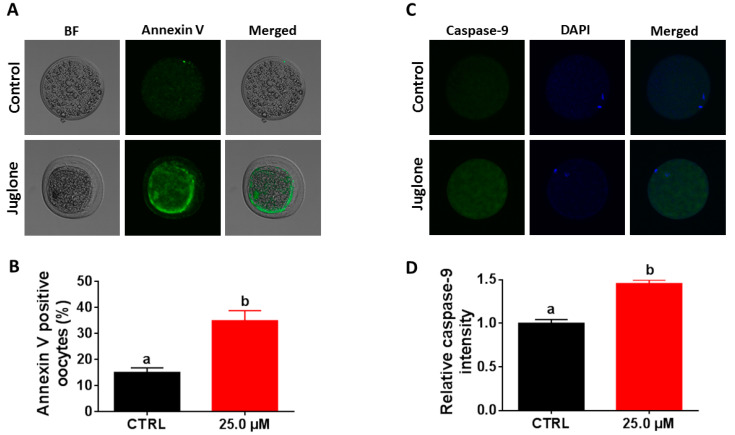
Effect of juglone on apoptosis of oocytes. (**A**) Oocytes post annexin V-staining. (**B**) Percentage of annexin V-positive oocytes. (**C**) Immunofluorescence of caspase-9 in untreated and juglone-treated (25.0 µM) oocytes. (**D**) Relative caspase-9 intensity in oocytes. Original magnification 100×. BF: bright field. The different letters on the columns reflect statistical significance (*p* < 0.05).

According to the above-mentioned data, the next experiments were executed using 25.0 µM of juglone in addition to the untreated control. Since juglone was able to induce apoptosis in several cell lines, the potential effect of juglone on apoptosis in oocytes was assessed by annexin V-labeling, as well as investigating the expression level of the apoptosis-specific protein caspase-9. As seen in Figure 6A,B, the percentage of annexin V-stained oocytes was higher in the juglone-treated group than control (*p* < 0.05). In addition, the results of immunofluorescence showed overexpression of caspase-9 in the 25.0 µM juglone-treated oocytes compared to the untreated control (Figure 6C,D; *p* < 0.05).

## 3. Discussion

Despite the use of juglone in traditional medicine as an anticancer and antimicrobial agent, little is known about its impact on embryonic development and the female reproductive system. In the present, we sought to understand the effect of juglone on the developmental competence of oocytes by using an in vitro model of bovine oocyte maturation. To attain this objective, the IVM medium was supplemented with different concentrations of juglone, while cumulus expansion, nuclear maturation, mitochondrial distribution, cleavage/blastocyst developmental rates, ROS, and apoptosis levels were investigated.

Morphological examination of COCs before and post-maturation showed a severe reduction in cumulus expansion after juglone treatment in a dose-dependent manner. In addition, the expression pattern of cumulus-specific genes, inspected using RT–qPCR, exhibited a significant downregulation of PTGS2, PTX3, TNFAIP6, and HAS2 in oocytes treated with 25.0 µM juglone, confirming the toxic effect of juglone on cumulus cells. Generally, the degree of cumulus expansion correlates with the competence of in vitro oocyte maturation that can eventually predict the developmental competence of embryos [31]. Similar to our observations, previous studies on porcine COCs showed a significant reduction in cumulus expansion and/or downregulation of HAS2, TNFAIP6, and PTX3 following treatment with juglone and the pro-oxidant molecules atrazine and triclosan [25,32,33].

Furthermore, the communication between cumulus cells and the oocyte, during maturation, significantly influences the subsequent oocyte’s developmental cascade [34]. Previously, the essential roles of maturation-related genes, including GDF-9, MARF1, DNMT1, and BMP-15, for the developmental competence of oocytes have been reported [35,36,37,38]. Although the RT–qPCR did not show a significant difference in the expression level of MARF1 and DNMT1, microscopic investigation of the nuclear material using DAPI-staining, generally used as an indicator for successful maturation, showed a significant reduction in the percentage of MII-oocytes, following juglone treatment in a dose-dependent manner. In line with our observation, Zhang et al. recently reported that 100 µM juglone treatment for 44 h during the IVM of porcine oocytes was associated with decreased maturation level, an effect that was neutralized upon vitamin C cotreatment [25]. Likewise, the toxicity of rotenone and malathion was able to decrease the maturation process [39,40]. Altogether, this highlights the negative influence of juglone on COC maturation.

Due to the critical role of oocyte maturation for the development of embryos, we moved forward to investigate if exposing COCs to juglone can also affect the rate of development. Juglone treatment decreased the rates of cleavage and 8–16 cell stage (evaluated at day 4) and blastocyst (evaluated at day 8), compared to the untreated control. These observations match with the results of oocyte maturation and confirm the detrimental effect of juglone on the overall process of embryo development. This also supports Zhang et al. that showed a significant decline in the developmental rate of early mouse embryos when juglone was administered post-fertilization at 10 and 50 µM concentrations, the effect that was abrogated upon treatment of vitamin C [30].

We went further to unravel the possible mechanisms behind the negative effect of juglone on maturation and subsequent embryonic development. Previously, several studies showed that embryonic development and the health of offspring could be influenced by oxidative stress and mitochondrial dysfunction in oocytes [23,41,42]. In this regard, the ROS are released as byproducts of cellular metabolic processes, yet, the excessive ROS release can induce cellular damage and apoptosis that can impair the proper oocyte maturation and the development of embryos [40,43]. Using the ROS measurement kit, higher ROS levels in juglone-exposed oocytes were observed that associated with the dosage of juglone, indicating its involvement in the induction of oxidative stress during IVM. Similarly, earlier studies revealed a significant accumulation of endogenous ROS and a depletion of glutathione (GSH) in human promyelocytic leukemia (HL-60), gastric cancer (SGC-7901), and ovarian cancer (OVCAR-3), as well as murine melanoma (B16F1) cells treated with juglone for 24–48 h [2,3,5,44], which confirms our finding in COCs. In addition, it corroborates with the recent study that showed increased levels of ROS in early mouse embryos treated with 10 µM juglone [30].

Because of the importance of mitochondria for the development of oocytes and embryos by providing energy in the form of ATP, we investigated the distribution pattern of mitochondria in oocytes following IVM. In previous reports, the high ROS levels were able to reduce mitochondrial function through disrupting mitochondrial distribution and membrane potential as well as ATP production [45]. Additionally, the distribution pattern of mitochondria in the cytoplasm during the maturation process is a critical indicator for the quality of mature oocytes, and hence, the difference in mitochondria localization is associated with differences in the developmental competence of oocytes [46]. Interestingly, a higher incidence of aberrant mitochondrial distribution pattern was noticed in juglone-treated oocytes, whereas most untreated oocytes displayed homogenously distributed mitochondria. For more clarification, the use of RT–qPCR indicated lower expression levels of the two mitochondria-specific genes ATPase6 and ATP5F1E in juglone-treated oocytes. Altogether, this confirms the direct involvement of high juglone concentrations in the severe reduction of maturation and meiosis regulation in oocytes through inducing mitochondrial dysfunction and ROS accumulation. This also affirms the earlier reports showing detrimental effects of juglone on mitochondrial distribution and membrane potential in porcine oocytes and in HL-60, SGC-7901 and SKOV3 cancer cells [2,3,25,47]. This also supports the aforementioned observations on maturation, total cleavage, and embryo developmental rates.

Since the linkage between apoptosis and reduced embryo development has been discussed in several studies, we moved forward to assess the levels of apoptosis following juglone treatment. Exposure of oocytes to toxic compounds significantly impaired the development of embryos through induction of ROS-derived oxidative stress, apoptosis, and cell death [32,33,48,49]. Staining of oocytes with annexin V showed a higher incidence of apoptosis after juglone treatment. In addition, the apoptosis-specific protein caspase-9 exhibited a greater abundance profile in juglone-exposed oocytes endorsing the apoptosis-inducing potential of juglone. This confirms previous studies that showed activation of caspase-3 and procaspase-9 in HL-60, SGC-7901, SKOV3, Hela, and B16F1 cells post-juglone treatment [2,3,5,47]. It also corroborates our above-mentioned records regarding juglone-induced damage in bovine oocytes as well as other studies documenting the induction of cellular apoptosis and reduction of oocyte quality during meiotic progression following the successive accumulation of ROS [32,39,40]. Collectively, these results indicated that juglone induces the accumulation of ROS, which in turn can reduce oocyte maturation and induce apoptosis, which can eventually impair the embryo development.

## 4. Materials and Methods

### 4.1. Chemicals and Reagents

All reagents were purchased from Sigma-Aldrich (St. Louis, MO, USA) unless otherwise specified.

### 4.2. Oocyte Isolation and IVM

The COCs were collected and cultured, as previously mentioned [50]. Briefly, COCs, aspirated from ovarian follicles using 18-gauge needles connected to 50 mL tubes containing TL-HEPES, were washed, and those comprising more than three cumulus layers were collected under a stereomicroscope (Olympus SZ51, Tokyo, Japan), washed three times in TL-HEPES, and cultured (around 50 COCs per well) in four-well plates containing 700 μL IVM medium (TCM-199 supplemented with 10% (*v/v*) fetal bovine serum (FBS), 10 μg/mL follicle-stimulating hormone (FSH), 1 μg/mL estradiol-17β, 0.2 mM sodium pyruvate, 10 ng/mL epidermal growth factor (EGF), 0.6 mM cysteine, 0.1 mg/mL streptomycin, and 100 IU/mL penicillin), in the absence or the presence of juglone (12.5, 25.0, and 50.0 μM) at 38.5 °C and 5% CO_2_ for 22 h. Checking the literature for juglone in vitro studies revealed that it could be used at concentrations in the range of 1.0–100.0 µM [25,30]. For preparing the three tested concentrations (12.5, 25.0, and 50.0 μM), juglone was first dissolved in DMSO then all the further dilutions were performed in IVM medium. The final DMSO concentrations in the medium were 0.25, 0.5, and 1.0% (for 12.5, 25.0 and 50.0 µM juglone, respectively). However, in a preliminary experiment, these concentrations did not show any significant impact on the development of blastocysts (Figure S1; *p* > 0.05).

### 4.3. In Vitro Fertilization (IVF) and Development of Blastocysts

The fertilization of oocytes was performed as previously described [23]. In brief, the frozen Hanwoo bull’s semen was thawed and diluted in warm DPBS (Dulbecco’s phosphate-buffered saline) then spun for 5 min at 1800 rpm. The pellet was suspended in 500 μL of 20 μg/mL heparin prepared in IVF medium (Tyrode’s lactate solution supplemented with 22 mg/mL sodium pyruvate, 6 mg/mL bovine serum albumin (BSA), 100 IU/mL penicillin, and 0.1 mg/mL streptomycin) and incubated at 38.5 °C for 15 min then subjected to dilution in IVF medium to a final concentration of 1–2 × 10^6^ spermatozoa/mL. Each COCs group was loaded with 700 μL of diluted sperm then incubated at 5% CO_2_ and 38.5 °C for 18–20 h.

Post-fertilization, successive pipetting was applied to detach cumulus cells, then the presumptive zygotes (Four replicates, *n* = 50) were washed and maintained in four-well plates containing 700 μL of complete SOF medium [51] before incubation at 5% CO_2_ and 38.5 °C. On day 4 after fertilization (day of fertilization was considered as day 0), the total cleavage and 8–16 cell-stage embryo development rates were estimated then the SOF medium was replenished, followed by incubation at 38.5 °C and 5% CO_2_ for 96 h. On day 8, blastocysts were visualized under stereomicroscope while the development rates of the treated and the control groups were recorded.

### 4.4. Estimation of Cumulus Cells Expansion and Oocyte Maturation

To assess the expansion process, COCs (Four replicates, *n* = 50) were morphologically inspected under an epifluorescence microscope (Olympus IX71, Tokyo, Japan) at the onset and the end of IVM. In brief, the COCs were initially visualized at day 0 and day 1 from the initial maturation, and then the number of COCs with normal expansion was counted. For the area of expansion, the surface area of each COCs was measured on day 1 using ImageJ software (National Institutes of Health, Bethesda, MD, USA; https://imagej.nih.gov/ij/) and subtracted from the values of day 0. For evaluating oocyte maturation, COCs (Triplicate, *n* = 30), collected after 22 h from the start of IVM and denuded by successive pipetting, were incubated with DAPI (4′,6-diamidino-2-phenylindole) to stain the nuclear material and visualized under a confocal laser-scanning microscope (Olympus Fluoview FV1000, Tokyo, Japan) where oocytes were classified either as metaphase II (MII; mature) or germinal vesicle stage (GV; immature).

### 4.5. Measurement of ROS Levels in Oocytes

At the end of the IVM step, oocytes (*n* = 20, four replicates) were denuded by successive pipetting and incubated with 5 μM H_2_DCFDA (2,7-dichlorodihydrofluorescein diacetate, a ROS indicator) for 20 min at 38.5 °C before washing thrice in PBS. Using an epifluorescence microscope, the stained oocytes were directly imaged under 490 nm excitation and 525 nm emission wavelengths, and the mean fluorescence intensity of individual oocytes was measured using ImageJ software.

### 4.6. Assessment of Mitochondrial Distribution Pattern in Oocytes

The distribution profile of mitochondria was investigated using Mito-Tracker deep Red FM (Molecular Probes, Eugene, OR, USA) as following. In brief, oocytes (*n* = 20, four replicates) were incubated with 100 nM Mito-Tracker Red stain for 40 min before washing in PBS and fixing in 4% paraformaldehyde. Oocytes were examined under an epifluorescence microscope where mitochondria were classified either as homogeneous (dispersed throughout the cytoplasm) or aberrant (distributed peripherally/semi-peripherally).

### 4.7. RNA Extraction and RT–qPCR

The extraction of RNA from COCs (*n* = 50, triplicate) was performed using an Arcturus PicoPure RNA isolation kit (Arcturus, Foster, CA, USA). The RNA was eluted in elution buffer then the concentration and the purity were estimated using NanoDrop 2000c spectrophotometer (Thermo Fisher Scientific, Waltham, MA, USA). The cDNA was synthesized using an iScript cDNA synthesis kit according to the manufacturer’s guidelines (Bio-Rad Laboratories, Hercules, CA, USA). In brief, 4 μL of 5X iScript reaction mixture, 15 μL of RNA (200 ng), and 1 μL of iScript reverse transcriptase were mixed and incubated at 25 °C for 5 min, 42 °C for 30 min, and 85 °C for 5 min.

The RT–qPCR was carried out using CFX96 instrument (Bio-Rad Laboratories) and iQ-SYBR Green Supermix (Bio-Rad Laboratories) as follows: 5 μL SYBR Green mix + 2 μL primer mix (Table 1) + 3 μL nuclease-free water-diluted cDNA were mixed and subjected to the following qPCR reaction conditions: 95 °C for 3 min and 44 cycles of 95 °C for 15 s, 58 °C for 20 s, and 72 °C for 30 s. Each cDNA sample was applied in duplicate, while relative expression was calculated using GAPDH as a reference gene and ΔΔCt method.

### 4.8. Annexin V Staining

Early apoptosis in oocytes was investigated using annexin V labeling according to the manufacturer’s instructions (Roche Diagnostics, Mannheim, Germany). Briefly, oocytes (Four replicates; *n* = 15–20) were denuded by repeated pipetting and washed three times in DPBS before incubation with Alexa Fluor 488-conjugated annexin V solution for 15 min at room temperature. Oocytes were washed and examined under a confocal laser-scanning microscope (488 nm excitation and 520 nm emission wavelengths), where the number of annexin V-positive oocytes was recorded.

### 4.9. Immunofluorescence

Mature oocytes (Four replicates; *n* = 20) were partially denuded by repeated pipetting followed by fixation with 4% paraformaldehyde. After three-times washing in PBS, oocytes were treated with 0.5% Triton X-100 for 30 min for permeabilization, then blocked with 3% BSA and 10% FBS for 2 h. Samples were incubated overnight at 4 °C with anti-Caspase-9 primary antibody (Sc-8355; Santa Cruz Biotechnology, Santa Cruz, CA, USA; diluted 1:200) [54], then washed thrice and incubated with Alexa Fluor 488-conjugated secondary antibody (A21206; Thermo Fisher Scientific, Waltham, MA, USA; diluted 1:500) for 90 min. The staining of the nuclei was performed via 15 min incubation with 1 ng/µL DAPI, and then oocytes were inspected under a confocal laser-scanning microscope (488 nm excitation and 520 nm emission wavelengths) while optical density was quantified by ImageJ software.

### 4.10. Statistical Analysis

The analysis of data was performed using GraphPad Prism version 6 (San Diego, CA, USA) using either Student’s *t*-test or one-way ANOVA combined with Tukey’s multiple comparisons. Data were tested for normal distribution and underwent a logarithmic transformation when this criterion was not met.

## 5. Conclusions

Collectively, our study reports that the administration of high juglone concentrations during the in vitro maturation can severely affect the development of oocytes and embryos through the direct induction of oxidative stress, apoptosis, and mitochondrial dysfunction. The study also indicates the potential negative effects of high juglone concentrations on oocyte maturation and embryo development when applied for therapeutic purposes. Finally, the interaction between juglone and the various cellular signaling pathways that regulate the different developmental stages of preimplantation embryos still needs to be addressed in future studies.

## Figures and Tables

**Table 1:** List of primer sequences.

Genes Names	Sequences	PCR Products (bp)	References
HAS2	F: GGATCTCCTTCCTCAGCAGTGT R: ATTCCCAGAGGTCCGCTAATG	106	[22]
TNFAIP6	F: TGAAAGATGGGATGCATATTGC R: CATTTGGGAAGCCTGGAGATT	101	[52]
PTGS2	F: CTTAAACAAGAGCATCCAGAATGG R: GCTGTACGTAGTCTTCAATCACAATCT	106	[52]
PTX3	F: CATGTATGTGAATTTGGACAACGA R: GCTTGTCCCACTCGGAGTTC	101	[52]
DNMT1A	F: ACGAATGGTGGATTGCTGGT R: CAGGTCTTCGTAGGTGGAGTC	197	[22]
MARF1	F: GCAGAGCACCAGGACAATCA R: GAAATAGCCCGCAGAGGAAG	262	[31]
ATPase6	F: GAACACCCACTCCACTAATCCCAAT R: GTGCAAGTGTAGCTCCTCCGATT	147	[53]
ATP5F1E	F: CAGGCTGGACTCAGCTACATC R: AGTCTTCATGGCGTTTGCTT	96	[23]
POLG2	F: CTTCTGGGAAACTACGGGAGAAC R: GTAGCCTCTTGTTTACCAGATCCA	84	[23]
GADPH	F: CCCAGAATATCATCCCTGCT R: CTGCTTCACCACCTTCTTGA	185	[50]

## Data Availability

The data presented in this study are available on request from the corresponding author.

## Supplementary Materials

The following are available online at https://www.mdpi.com/1422-0067/22/1/168/s1, Figure S1: Effect of DMSO on blastocyst development.
