# Supplementary figures and images for: Induction of Oxidative Stress and Mitochondrial Dysfunction by Juglone Affects the Development of Bovine Oocytes
Source: Int J Mol Sci. 2020 Dec 26;22(1):168. doi: 10.3390/ijms22010168 (PMC7794829; doi:10.3390/ijms22010168)

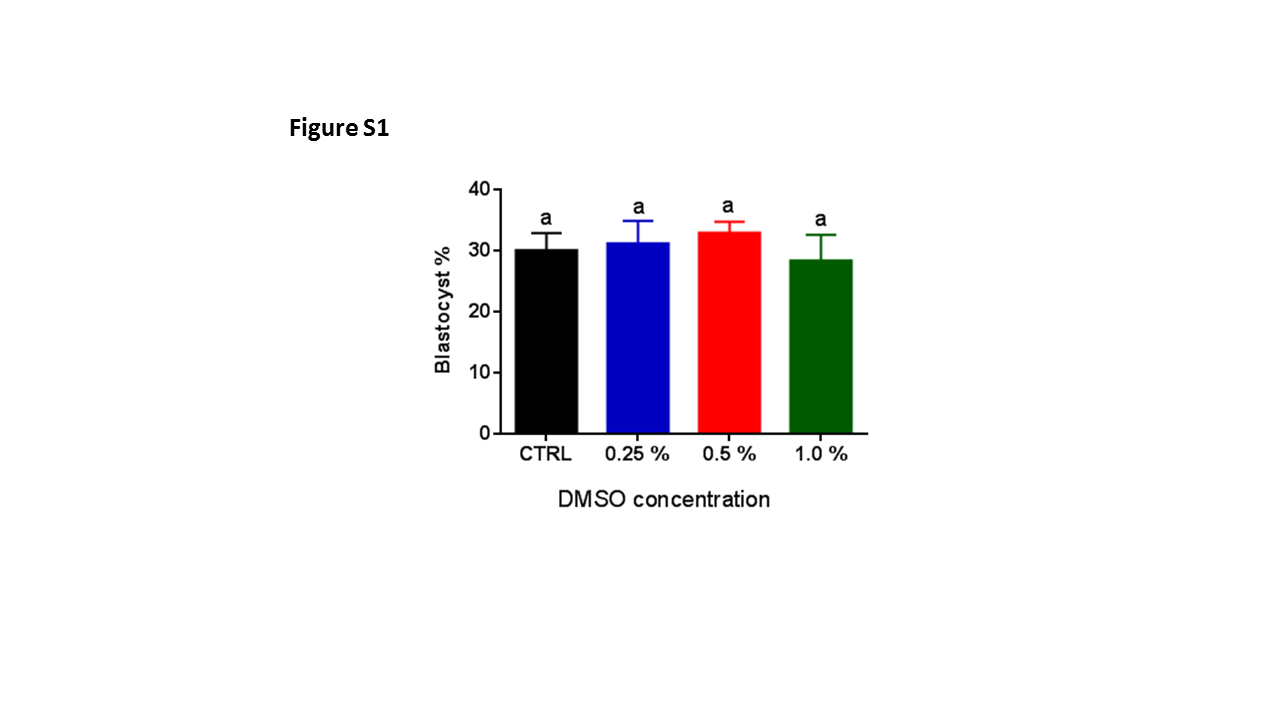

Supplement: Supplementary file 1 [file ijms-22-00168-s001.zip › ijms-1061107-supplementary.TIF]
